# Sickle cell retinopathy molecular investigation through proteomic analysis of Townes HbSS mice retinas

**DOI:** 10.3389/ebm.2026.11228

**Published:** 2026-10-14

**Authors:** Júlia Nicoliello Pereira de Castro, Ana Rafaela Carvalho Monte, Ana Carolina Rodrigues de Oliveira, Julia Dezordi Acacio, Thiago Adalton Rosa Rodrigues, Mirella Telles Salgueiro Barboni, André Maurício Passos Liber, Daniela Maria Oliveira Bonci, Dora Fix Ventura, Fernando Ferreira Costa, Ana Carolina Lima Camargo, Mônica Barbosa de Melo

**Affiliations:** 1 Laboratório de Estudos em Genética Humana, Centro de Biologia Molecular e Engenharia Genética (CBMEG), Universidade Estadual de Campinas - UNICAMP, Campinas, Brazil; 2 Department of Ophthalmology, Semmelweis University, Budapest, Hungary; 3 Institute of Molecular and Clinical Ophthalmology Basel (IOB), Basel, Switzerland; 4 Université Paris-Saclay, CNRS, Institut des Neurosciences Paris-Saclay, Saclay, France; 5 Departamento de Psicologia Experimental, Universidade de São Paulo, São Paulo, Brazil; 6 Centro de Hematologia e Hemoterapia, Universidade Estadual de Campinas - UNICAMP, Campinas, Brazil

**Keywords:** metabolism, phototransduction, proteomics, sickle cell retinopathy, Townes HbSS

## Abstract

Sickle cell retinopathy (SCR) is the most important ocular manifestation in sickle cell disease (SCD). Retinal tissue is highly sensible to vaso-occlusion and hypoxia observed in SCR pathophysiology, with consequent vessel remodeling and ultimately neovascularization in its proliferative form. Severe cases are followed by vitreous hemorrhage and retinal detachment, with 10–20% of vision loss in affected eyes. Current treatment options such as anti-VEGF (Vascular Endothelial Growth Factor) injections and laser photocoagulation are based on other proliferative retinopathies, without pre-clinical and clinical studies specific for SCR. To gain insights into disease-associated molecular pathways and to support the identification of candidate biomarkers and therapeutic targets, we performed comparative proteomic analysis in retinas of Townes HbSS mice, a humanized mouse model of sickle cell anemia (SCA), and C57BL/6J as controls. Blood hematological and metabolic features, retinal activity, and morphology were characterized. Comparative label-free proteomic analysis of retinas from Townes HbSS (N = 5) and control (N = 7) groups was performed in a LTQ Orbitrap Velos equipment, followed by functional enrichment and interaction analysis. The Townes HbSS group presented blood parameters consistent with the SCA phenotype, and retinal degeneration was indicated by functional and morphological alterations in external layers. Pathways of energetic metabolism and phototransduction were enriched among the downregulated proteins, and the molecular downregulation profile of Cnga1 and Slc24a1, related to photoreceptor activation, was validated by RT-qPCR. These findings highlight hypoxia-associated molecular adaptations in metabolic respiration and phototransduction linked to retinal degeneration. Taken together, our results contribute to a more comprehensive characterization of the molecular landscape underlying sickle cell retinopathy.

## Impact statement

This study presents a comprehensive proteomic characterization in retinas of Townes mice, a sickle cell anemia humanized mice model. The differentially expressed proteins obtained reveals relevant pathways for sickle cell retinopathy (SCR) progression, such as phototransduction and metabolic pathways of oxidative phosphorylation and citric acid cycle. These findings provide a better understanding of the molecular mechanisms of SCR pathophysiology, prior to more evident functional and morphological alterations. Treatment options specific for SCR are scarce and based in other retinopathies. Thus, this work supports further studies aiming to the identification of biomarkers and the development of mechanism-based therapeutic strategies.

## Introduction

Sickle cell disease (SCD) is a group of hereditary hemoglobinopathies with significant global health burden [1] characterized by a single point mutation in the sixth codon of the β-globin gene (c.20A > T). The replacement of glutamic acid for valine (Glu6Val) culminates in the synthesis of HbS, a hemoglobin variant. Mutations in both alleles of the gene lead to the sickle cell anemia (SCA) phenotype. In addition, the HbS may be associated with other hemoglobin variants, such as in the Hemoglobinopathies SC (HbSC), SD (HbSD) and S/βTal. The main feature of HbS is the polymerization when deoxygenated, with consequent erythrocyte sickling. The altered format and rigidity of the red cell membrane facilitate hemolysis, endothelial dysfunction, vaso-occlusion and ischemia, with a myriad of acute and chronic complications [2].

These alterations in red blood cells are harmful to several tissues, particularly the retina, which has a high metabolic demand [3]. In this context, sickle cell retinopathy (SCR) is the most important ocular manifestation in SCD. Goldberg classification [4] divides SCR in non-proliferative (NPSCR) and proliferative (PSCR). Vaso-occlusion and hypoxia of the peripheral microvasculature and vessel remodeling are found in NPSCR. In PSCR, neovascularization occurs in response to the hypoxic environment. It is known that spontaneous vessel regression can occur in 20%–60% of cases. However, new blood vessels are fragile and prone to rupture, and, in advanced stages, can lead to vitreous hemorrhage and ultimately to retinal detachment [5], with vision loss in 10%–20% of the affected eyes [6].

There is an inverse relationship between SCD genotypes and the prevalence and severity of SCR. SCA patients present severe multiorgan damage but are less affected by SCR (2.6%). In contrast, HbSC patients have a milder systemic condition, but a higher prevalence of SCR (32,8%), especially in its more aggressive form, PSCR [2]. Although increased hematocrit and blood viscosity in HbSC patients may partially account for the higher frequency of SCR [7], this hemodynamic explanation does not fully resolve the inverse association between systemic severity and retinal involvement. This clinical–biological dissociation indicates the contribution of genotype-specific molecular mechanisms that remain insufficiently characterized.

A better understanding of these molecular mechanisms involved in SCR progression is essential to the development of earlier, more specific, and effective treatment strategies. Currently, there are no established therapeutic options for NPSCR beyond clinical monitoring. For PSCR, laser photocoagulation, intravitreal injections of Vascular Endothelial Growth Factor antibodies (anti-VEGF), and vitrectomy are the main therapeutic approaches. However, these interventions are invasive, associated with potential side effects, risk of neovascularization, and lack prospective clinical trials specifically focused on SCR context [5].

Proteomic analysis is a robust technique, capable of absolute and relative quantification of protein expression profiles with cellular and tissue specificity, enabling the identification of markers in various disease contexts. In diabetic retinopathy (DR) and retinopathy of prematurity (ROP), proteomic studies have identified targets related to inflammation, cell adhesion, proliferation, oxidative stress, and energy metabolism, pathways known to contribute to the development of proliferative retinopathies [8, 9]. However, a comprehensive evaluation of the molecular mechanisms in SCR remains scarce. In this original study, we performed proteomic analysis of retinas from Townes HbSS mice, a humanized mouse model of sickle cell anemia. The analysis of differential protein expression in SCR retinas may provide insights into disease-associated molecular pathways, contributing to further studies, aiming to improve prognostic stratification and the development of mechanism-based therapeutic strategies for this SCA complication.

## Methods

### Animals

All animal experimental procedures and protocols were carried out according to the ARVO Statement for the Use of Animals in Ophthalmic and Vision Research. The protocol was approved by the Ethics Committee on the Use of Animals of the State University of Campinas (Protocols 6131-1/2022; 6131-1(A)/2023; 6392-1/2024) and followed the Ethical Principles in Animal Experimentation validated by the Brazilian College of Animal Experimentation. Adult female C57BL/6J and transgenic Townes HbSS mice (JAX Strain #:013071) 8 months old, were kept under controlled temperature conditions (22–25 °C), relative humidity (55%), 12-h photo period and free access to water and standard diet. The number of animals used is described in the figure legend of each correspondent result.

### Electroretinogram (ERG)

The electroretinography (ERG) was performed in both experimental groups, C57BL/6J and Townes HbSS, using the Ganzfeld stimulator (Q450SC, Roland Consult) coupled to the RetiPort System (Roland Consult, Brandenburg, Germany). The technique detects variations in electrical potential between the cornea and the retina in response to light stimuli of different intensities. Prior to binocular ERG recordings, the animals were dark-adapted for at least 12 h and then anesthetized with an intraperitoneal (i.p.) injection of ketamine hydrochloride (80 mg/kg) and xylazine hydrochloride (10 mg/kg). The pupils were fully dilated with 1% tropicamide (Mydriacyl®; Alcon, São Paulo, Brazil) and 2% methylcellulose (Ophthalmos®; São Paulo, Brazil) was used to prevent corneal injury. Goldring electrodes (Ø 1 mm; Roland Consult, Brandenburg, Germany) were positioned on the corneas (active) and needle electrodes (Roland Consult, Brandenburg, Germany) placed subcutaneously medial to the two ears (reference) and at the base of the tail (ground electrode). All signals were amplified 100,000 times, filtered with a bandpass filter between 1 and 300 Hz, and digitized at a rate of 512 Hz. During recordings, mouse body temperature was maintained at 38 °C using a water-heated platform. All preparation and electrode placement was conducted under dim red light.

The ERG protocol was an extended version of the ISCEV protocol (International Society for Clinical Electrophysiology of Vision). Protocols and procedures have been described in detail elsewhere [10–12]. Briefly, under anesthesia, dark-adapted responses (rod and mixed rod-cone response) were recorded to flashes of −3.8, −2.8, −1.8, −0.8, and 0.2 log cd·s/m^2^ (white light) strengths on a dark background. The number of repeats (sweeps) decreased with increasing flash strength (12, 10, 8, 8, and 4, respectively). The inter-stimulus interval (1, 2, 5, 10, and 20 s) and the interval between conditions (10–120 s) were increased accordingly to maintain the dark-adapted state. The light-adapted response (cone response) was recorded using flashes of 0.3 log cd·s/m^2^ on a background of 25 cd/m^2^ (white light) after 2 min of light adaptation. Twenty flashes were averaged with 1 s inter-stimulus interval.

ERG components from both eyes were analyzed by detecting the troughs and peaks of the a- and b-waves using the MATLAB program routines (The MathWorks Inc., Natick, Massachusetts, USA), written and kindly provided by Professor Jan Kremers (University Hospital Erlangen, Erlangen, Germany) and with Excel spreadsheets (Microsoft Office 2010, © Microsoft Corporation, Redmond, WA, USA). The oscillatory potentials (OPs) were isolated and removed from the scotopic recordings by a variable filter method [13]. ERGs without OPs were used to measure a- and b-wave parameters. Isolated OPs were also analyzed. The amplitude of the scotopic a-wave was defined as the difference in μV between the baseline (average of recordings 16 ms before the occurrence of the flash) and the minimum within 50 ms after stimulus onset. The amplitude of the scotopic and photopic b-wave was the difference in μV between a-wave trough and the peak of the b-wave. The implicit times corresponded to the intervals between the stimulus onset and the peaks of the a-wave and of the b-wave. Abnormal responses and aberrant signals were excluded after inspection of the raw signal from each animal. The photopic a-waves presented very small amplitudes and were therefore not considered in the analysis.

Next, euthanasia was performed via intraperitoneal injection of ketamine hydrochloride (240 mg/kg) and xylazine hydrochloride (30 mg/kg), followed by enucleation of both ocular globes. The retinas of the left ocular globes were isolated for molecular analyses, and the right ocular globes were subjected to morphological analysis.

### Hematological and metabolic parameters

Blood collection was performed after euthanasia using EDTA tubes (MiniCollect® - EDTA K3, 1mL, Vacuette). The hematological parameters included red blood cells (RBC), hemoglobin (HB), hematocrit (HCT), white blood cell (WBC), platelets (PLT) and reticulocytes using Sysmex XN-Series XN™ (Roche, XN). The metabolic evaluation consisted in lactate dehydrogenase (LDH) and bilirubin quantification in the AU5800 Series™ (Beckman-Coulter) equipment.

### Hematoxylin-eosin staining

The ocular globe was fixed in Davidson’s solution (2% formaldehyde, 35% ethanol, 10% acetic acid diluted in water) for 24 h. The tissue was then dehydrated, and 5 µm sections were produced in a rotating microtome (Leica, RM2125, Germany) and collected on silanized slides. The slides were stained with hematoxylin and eosin (HE) for general morphology.

### Protein extraction and proteomic analysis

A pool of 2 retinas of different animals per sample were dissected with lysis buffer (Urea 8M, Tris-HCl 1M pH 7.2, PMSF 100nM, dithiothreitol (DTT) 65 mM and cOmplete™ Mini (Merck # 11836153001) protease inhibitor cocktail) in a PowerLyzer 24 homogenizer (Qiagen). Samples were centrifuged (4 °C, 10,000 rpm for 20 min) and the supernatant was collected. Total protein concentration was determined by Bradford protein assay [14].

For protein digestion, samples were treated with 8M urea (U0631, Sigma-Aldrich), followed by protein reduction with DTT (D0632, Sigma-Aldrich), 5 mM for 25 min at 56 °C, and alkylation with iodoacetamide (IAA) (I6125, Sigma-Aldrich), 14 mM for 30 min at room temperature in the dark. For protein digestion, urea was diluted to a final concentration of 1.6 M with 50 mM ammonium bicarbonate (A6141, Sigma-Aldrich), and 1 mM of calcium chloride (C4901, Sigma-Aldrich) was added to the samples. For trypsin digestion, 2 ng/uL of trypsin (Sequencing Grade Modified Trypsin—V5111—Promega) was added. The samples were incubated for 16 h at 37 °C. The reaction was quenched with 1% formic acid (100264, Merck), and peptides were desalted with C18 stage tips (Octadecyl C18-bonded sílica—3 M Empore™ extraction disks), dried in a vacuum concentrator, reconstituted in 0.1% formic acid (100264, Merck), and stored at −20 °C for subsequent analysis by LC-MS/MS.

An aliquot of each pool of samples was loaded onto an LTQ Velos Orbitrap mass spectrometer (Thermo Fisher Scientific, Waltham, MA, USA) connected to a nanoflow LC (nLC-MS/MS) by an EASY-nLC system (Proxeon Biosystems, West Palm Beach, FL, USA) through a Proxeon nanoelectrospray ion source. The peptides were separated by a 2%–90% acetonitrile (ACN) gradient in 0.1% formic acid using an analytical column PicoFrit Column (20 cm × ID75 mm, 5 mm particle size, New Objective, Woburn, MA) at a flow of 300 nL/min over 212 min. The nanoelectrospray voltage was set to 1.7 kV, and the source temperature was 275 °C. All instrument methods for the LTQ-Orbitrap Velos were set up in the data-dependent acquisition mode. Full-scan MS spectra (m/z 300–1,600) were acquired by the Orbitrap analyzer after accumulation to a target value of 1 × 10^-6^. The resolution of the Orbitrap was set to r = 60,000, and the 20 most intense peptide ions with charge states ≥2 were sequentially isolated to a target value of 1,000 and fragmented in the linear ion trap by low-energy collision-induced dissociation (CID) as the dissociation or fragmentation method, with a normalized collision energy of 35%. The signal threshold for triggering an MS/MS event was set to 500 counts. Dynamic exclusion was enabled with an exclusion size list of 500, an exclusion duration of 30 s and a repeat count of 1. An activation q = 0.25 and an activation time of 10 ms were used.

### Data processing and analysis

LC-MS/MS runs were conducted for each region raw data using MaxQuant v1.3.0.3 software. MS/MS spectra were searched against The Mouse UniProt database using the Andromeda search engine. As search parameters, a tolerance of 10 ppm was considered for precursor ions (MS search) and 1.0 Da for-fragment ions (MS/MS search), with a maximum of two missed cleavages. Carbamidomethylation of cysteine was considered a fixed modification, and oxidation of methionine and protein N-terminal acetylation were considered variable modifications. A maximum of a 1% false discovery rate (FDR) was set for both the protein and peptide identification. Protein quantification was performed using the LFQ algorithm implemented in MaxQuant software with a minimal ratio count of 1 and a window of 2 min for matching between runs. The global normalization was used in the MaxQuant LFQ algorithm for label-free quantification (LFQ) [15]. Statistical analysis was performed with Perseus v1.6.7.0 software, which is available in the MaxQuant package. Identified protein entries were processed, excluding reverse sequences, “only by site” entries, and contaminants. Raw LFQ values were log transformed, and the mean of normalized intensities were compared between groups. Supplementary Table S6 lists all proteins along with their corresponding FDR and p-values. Since no protein reached significance under the FDR cutoff, proteins with p < 0.05 were selected for subsequent analysis, allowing an exploratory overview of biologically meaningful candidates. The mass spectrometry proteomics data have been deposited to the ProteomeXchange Consortium via the PRIDE partner repository [16] with the dataset identifier PXD082336.

### Functional enrichment and protein-protein interaction network

To better understand the biological relevance of the differentially expressed proteins (DEPs), the complete list was separated into upregulated (Log2Fold Change >0) and downregulated Log2Fold Change <0) for functional enrichment and protein-protein interaction (PPI) network construction. KEGG molecular pathway enrichment was performed using ShinyGO (p < 0.05) [17]. Next, String DB database [18] (confidence interval = 0.7) was used to construct the PPI network for DEPs present in the most relevant enriched pathways.

### Total RNA extraction and quantitative reverse transcription polymerase chain reaction (RT-qPCR)

To further evaluate the molecular expression profile of relevant DEPs, RT-qPCR was performed. Total RNA was extracted from the isolated retinas with the RNeasy® Mini kit (Qiagen, 74104, Germany), followed by purification with DNAse I Amplification Grade (Invitrogen, USA) to remove remaining genomic DNA. The quantity and purity of the extracted RNA were measured using NanoDrop2000 spectrophotometer (Thermo Scientific). Two micrograms of RNA were reverse transcribed with the High-Capacity Kit RNA-to-cDNA (Life Technologies, 4387406, Carlsbad, CA, USA). All the protocols followed the manufacturer’s instructions.

Next, the RT-qPCR reaction was performed using the SYBR Green PCR Master Mix® (Applied Biosystems, Foster City, CA, USA) detection method in an ABI StepOne Plus equipment (Applied Biosystems, Foster City, CA, USA). Specific primers were used for *Slc24a1* and *Cnga1* genes (Table 1). Briefly, the PCR amplification was carried out in a 10 μL reaction volume containing 2 μL of cDNA (1:10 dilution) and 8 μL of reaction mix, containing 200 or 400 nM of specific primers diluted in injection water and 3.15 μL of SYBR Green PCR Master Mix. All samples were run in duplicate with the following cycling conditions: 1 cycle of 95 °C for 10 min, 40 cycles of 95 °C for 15s, and 40 cycles of 60 °C for 1 min. Relative quantification of each gene was performed using the 2^-ΔΔCT method, according to Livak et al. [19]. The Ct values obtained for each sample were normalized by the ratio of the target gene and the beta-actin (*Actb*) and glyceraldehyde-3-phosphate dehydrogenase (*Gapdh*) reference genes mean value. Results were obtained by the expression ratio of the Townes HbSS/C57BL/6J groups.

**TABLE 1:** Primers used for RT-qPCR.

Gene	Primer sequence (5′-3′)	Amplicon length	Concentration
*Slc24a1*	F -TGTCAAGGTCTGAAGGTTTGG R - GGCTGCTCTGGGATTCATGT	88 pb	200 nM
*Cnga1*	F - CGGATGGAAAATGGAGCGTG R - AAGTGCTGCTCCCTCTGTGA	137 pb	200 nM
*Actb*	F - TCCAGCCTTCCTTCTTGGGTAT R - CGGATGTCAACGTCACACTT	85bp	400 nM
*Gapdh*	F - GCGGCACGTCAGATCCA R - CATGGCCTTCCGTGTTCCTA	55 pb	400 nM

F: forward sequence; R: reverse sequence.

### Statistical analysis

Statistical analysis was performed in GraphPad Prism® (version 10.00, GraphPad, Inc., San Diego, CA). The Shapiro-Wilk normality test was applied in all data. Two-way ANOVA was used for the ERG scotopic data, and Mann–Whitney test for the ERG photopic data. RT-qPCR, hematological and metabolic parameters were compared by Student’s *t*-test. All values were considered statistically significant when *p* < 0.05.

## Results

### Townes HbSS mice reproduced SCA hematological and metabolic phenotype

To confirm the SCA phenotype, hematological parameters were evaluated in Townes HbSS and C57BL/6J. The Townes HbSS mice showed hematological features consistent with disease, including a reduction of 58.23% in erythrocyte count (5.1 ± 0.9 and 8.8 ± 0.9 10^6^/uL); 52.10% in hematocrit (24.4 ± 4.1 and 46.9 ± 5.3%) and 46.49% in hemoglobin concentration (5.9 ± 1 and 12.6 ± 1.3 g/dL), in addition to expressive raise of 998% in reticulocyte count (41 ± 6.7 and 4.1 ± 1.1%) and of 702.33% in white blood cell count (30.5 ± 7.8 and 4.3 ± 2.1 10^3^/uL), all comparisons with p < 0.0001 (Figure 1).

**FIGURE 1:**
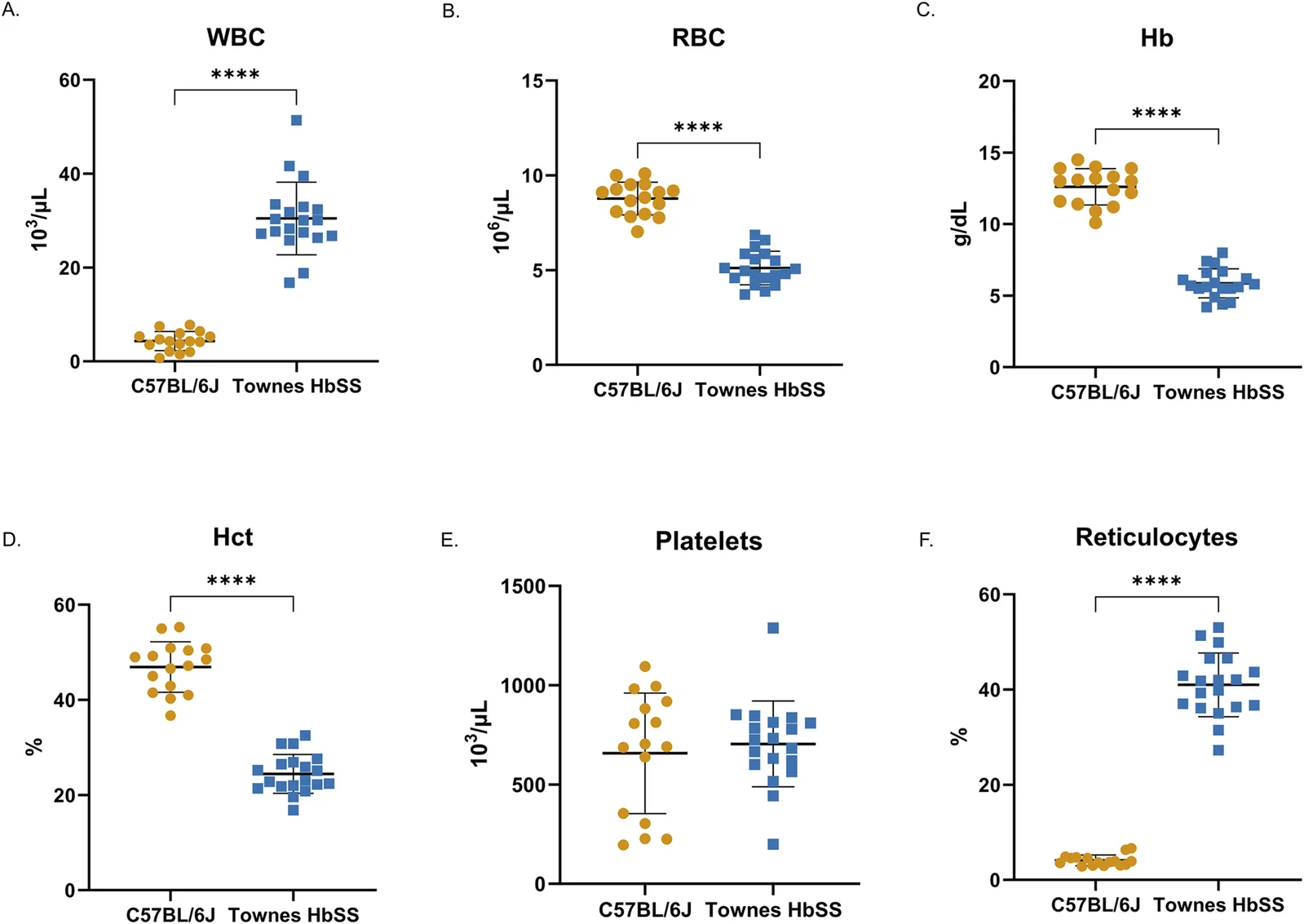
Hematological parameters of Townes HbSS and C57BL/6J mice. **(A)** WBC: total leukocyte count; **(B)** RBC: total erythrocyte count; **(C)** Hb: hemoglobin; **(D)** Hct: hematocrit. **(E)** Platelets; **(F)** Reticulocytes. ****Student’s t-test (****p < 0.0001) (N= C57BL/6J = 16/*Townes HbSS* = 19).

Regarding the evaluation of hemolysis markers, Townes HbSS group showed a significant increase of 852.83% in total bilirubin (1.4 ± 0.3 and 0.2 ± 0.02 mg/dL); 1,095.75% in direct (0.4 ± 0.1 and 0.03 ± 0.00 mg/dL) and 784.43% in indirect bilirubin (0.1 ± 0.2 and 1 ± 0.02 mg/dL), all comparisons with p < 0.0001, with no statistically significant difference in LDH levels between groups (Figure 2).

**FIGURE 2:**
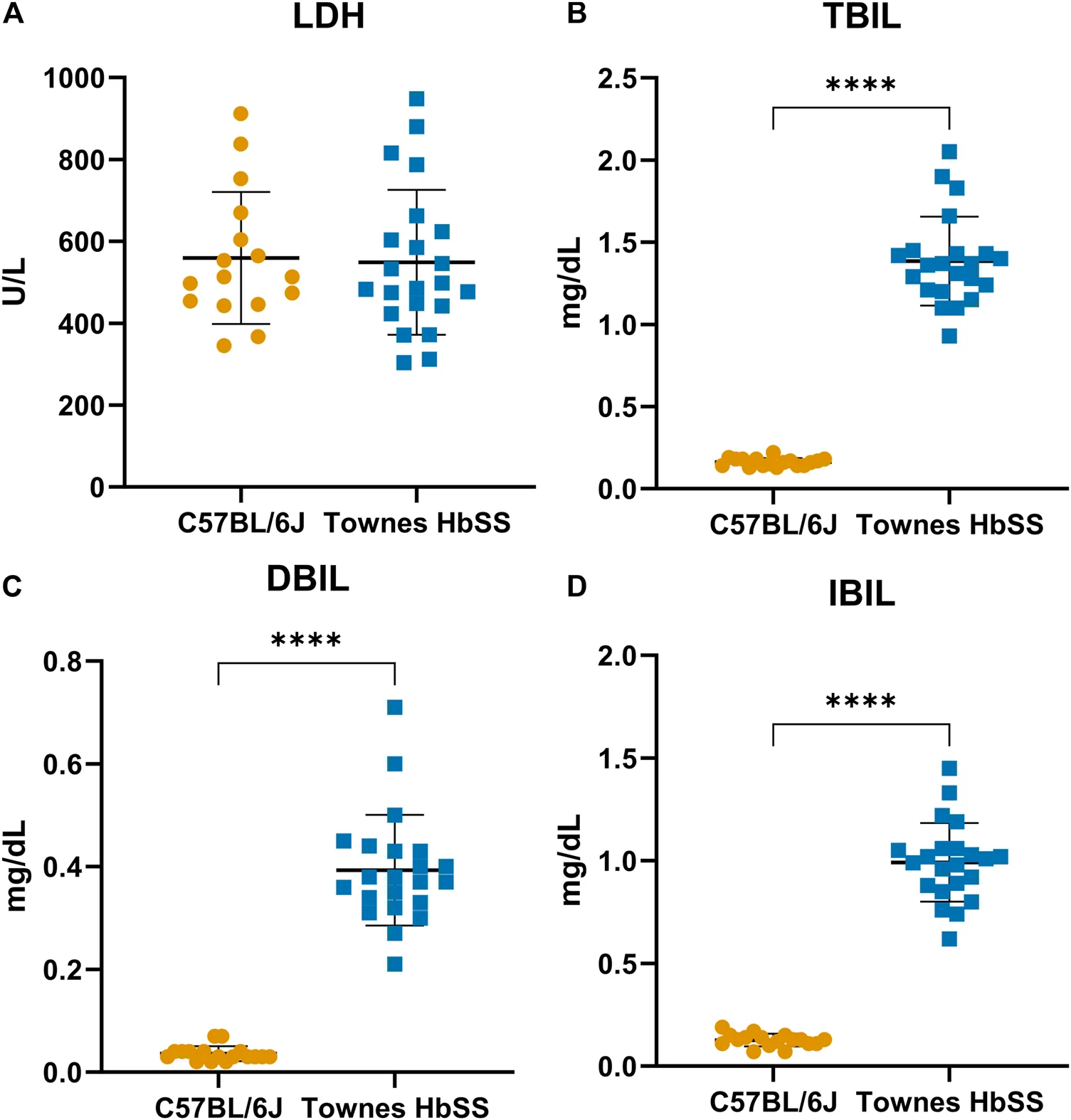
Hemolysis markers of Townes HbSS and C57BL/6J mice. **(A)** LDH: lactate dehydrogenase; **(B)** TBIL: total bilirubin; **(C)** DBIL: direct bilirubin; **(D)** IBIL: indirect bilirubin. ****Student’s t-test (****p < 0.0001) (N= C57BL/6J = 16/*Townes HbSS* = 22).

### Townes HbSS mice exhibited functional and morphological alterations in retinal layers

Retinal responses to light stimuli were evaluated by electroretinography (ERG) in all animals. Under scotopic conditions (Figure 3A), for the a-wave, referring to photoreceptor activity, the Townes HbSS mice showed smaller amplitude (153 ± 59.4 µV) when compared to the controls (182.8 ± 62.8 µV), presenting a reduced response to the stimulus at the highest light intensity (0.3 log cd·s/m^2^) (p = 0.016) (Figure 3B). A significant reduction was also observed in Townes HbSS implicit time in −0.7 log cd·s/m^2^ light intensity (20.1 ± 1.20 ms) compared to controls (20.82 ± 1.33 ms) (p = 0.0095) (Figure 3D). No statistically significant differences were observed between the experimental groups in either b-wave amplitude (Figure 3C) or implicit time (Figure 3E), which are mainly attributable to bipolar cell activity. Finally, compared to C57BL/6J (23.32 ± 7.18 µV), Townes HbSS mice (20.34 ± 7.49 µV) also exhibited reduced amplitude of OPs at maximum light intensity (0.3 log cd·s/m^2^) (p = 0.0274), (Figures 3F,G), indicating an early impairment of the inner retina.

**FIGURE 3:**
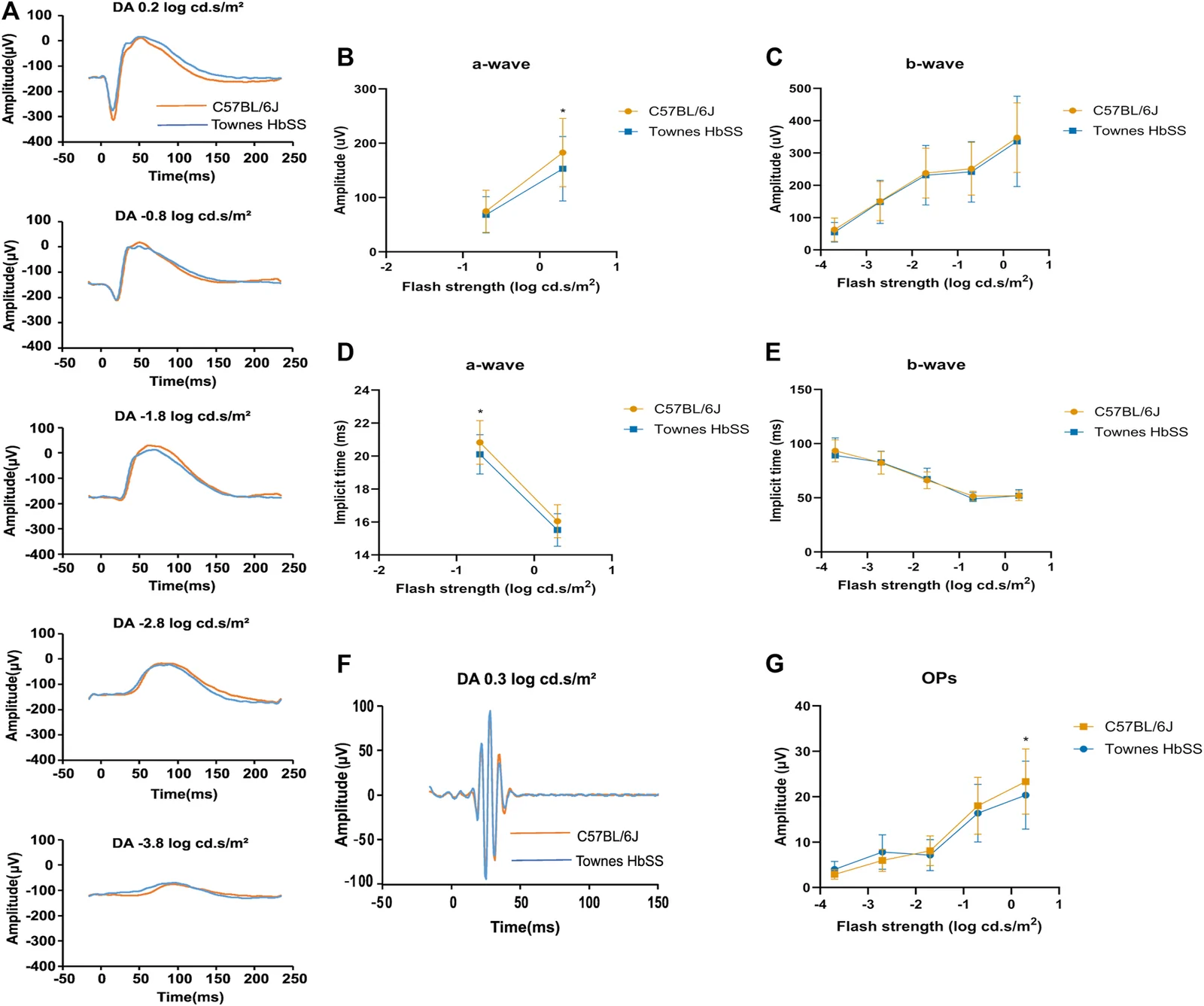
ERG dark-adapted responses of Townes HbSS (N = 38 eyes/19 animals) and C57BL/6J (N = 44 eyes/22 animals). **(A)** Average dark-adapted (scotopic) flash ERGs in C57BL/6J (orange traces) and Townes HbSS mice (blue traces). **(B,C)**: amplitude; **(D,E)**: implicit time; **(F)** Average oscillatory potentials traces isolated from the strongest scotopic flash in C57BL/6J (orange trace) and Townes HbSS (blue trace). **(G)** amplitude of oscillatory potentials. Results are shown as mean (± standard deviation). Two-way ANOVA. (*p < 0.05). µV, microvolts; ms, miliseconds; cd.s/m^2^, cd second per square meter.

For the photopic response, qualitative evaluation of the averaged responses of both groups shown in Figure 4A indicates that Townes HbSS showed a reduced amplitude of the photopic negative response (PhNR), possibly related to innermost retina alterations, specifically the ganglion cells layer. There was no significant difference in the median b-wave amplitude between Townes HbSS (69.69, 49.87-95.70 μV) and C57BL/6J (73.04, 50.74-97.07 μV) (Figure 4B). However, Townes HbSS (38.24, 36.63-39.49 ms) showed a higher implicit time of the b-wave compared to controls (37.21, 35.67 – 38.10 ms) (p = 0.0096), as shown in Figure 4C, indicating a delayed response in bipolar cells.

**FIGURE 4:**
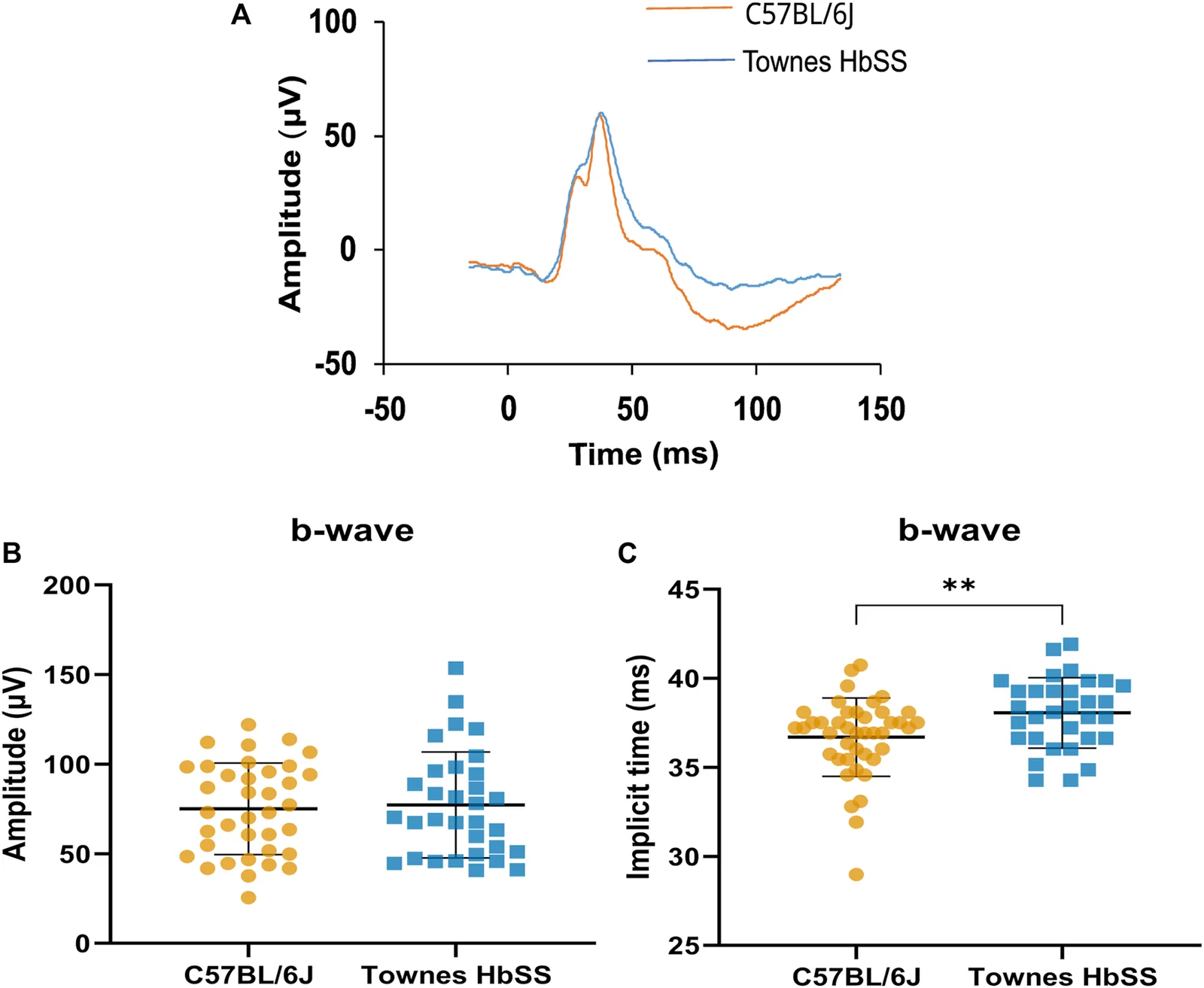
ERG light-adapted responses of Townes HbSS (N = 23/46 eyes) and C57BL/6J (N = 17/34 eyes). **(A)** Averaged light-adapted (photopic) flash ERGs in C57BL/6J (orange traces) and Townes HbSS mice (blue traces); **(B)** amplitude and **(C)** implicit time. Results are shown as median (first quartile Q1 - third quartile Q3). Mann-Whitney (**p < 0.001). µV, microvolts; ms, milliseconds; cd.s/m^2^, cd second per square meter.

In the HE staining, qualitative assessment of retinal morphology indicated disruptions in the outer nuclear layer (ONL) in C57BL/6J and Townes HbSS mice (Figure 5). Discrete alterations are observed in the inner nuclear layer (INL) in Townes HbSS sections (Figure 5B), findings that may be related to retinal degeneration. In addition, blood vessels were observed in the ganglion cell layer (GCL) in Townes HbSS compared to controls, as illustrated by the black arrow in Figure 5B.

**FIGURE 5:**
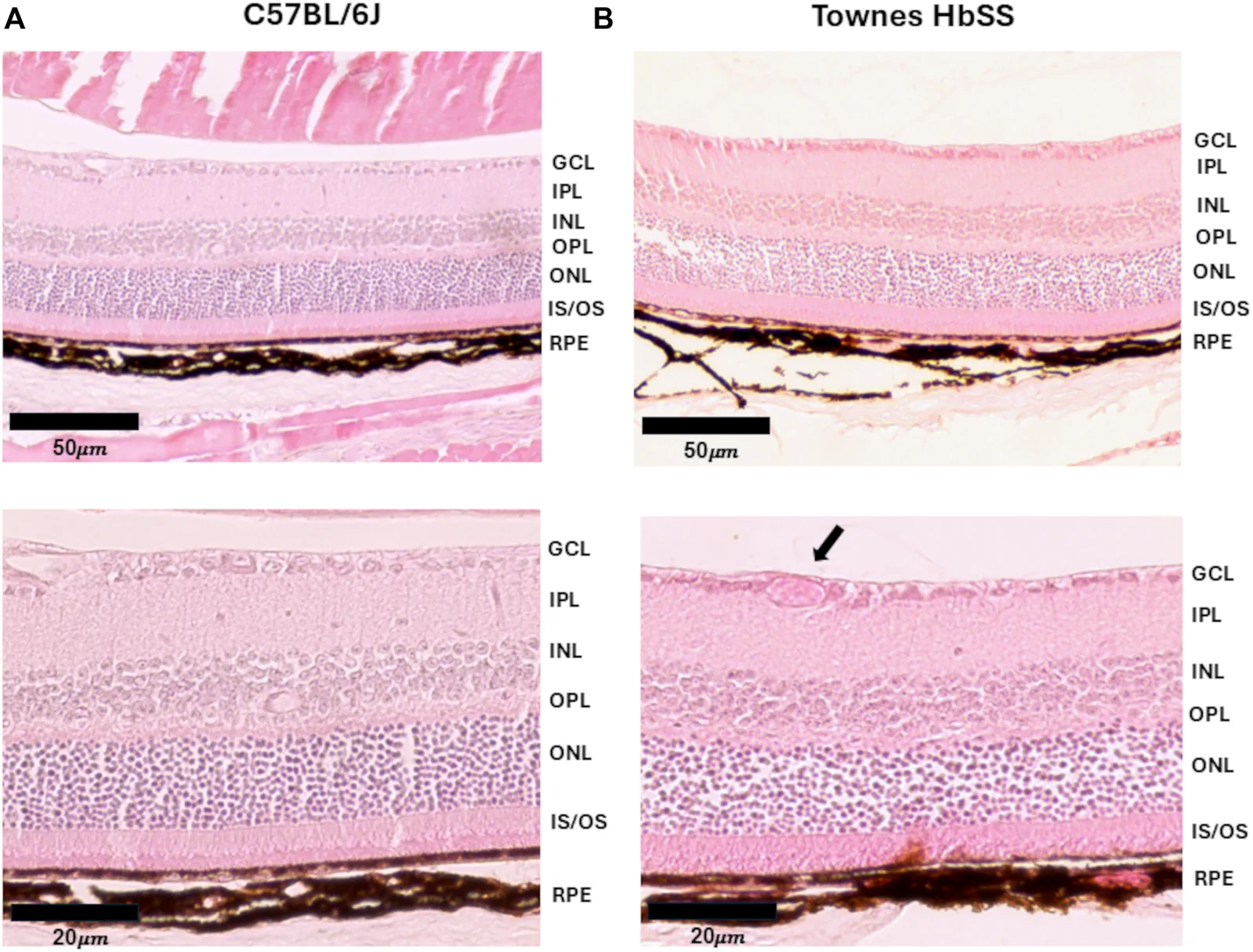
Illustrative images of Townes HbSS and C57BL/6J H&E sections. **(A)** C57BL/6J section in 20x (upper left) and 40x (down left) objectives. **(B)** Townes HbSS section in 20x (upper left) and 40x (down left) objectives. The black arrow indicates a blood vessel. GCL, Ganglion cell layer; IPL, Inner plexiform layer; INL, Inner nuclear layer; OPL, Outer plexiform layer; ONL, Outer nuclear layer; IS/OS, Inner segment/outer segment; RPE, Retinal pigment epithelium.

### Townes HbSS retinal proteomic landscape

Comparative proteomic profiling was performed to compare the retinal protein expression patterns between Townes HbSS mice and control animals. The mice retina proteome encompassed 2,248 proteins, of which 163 were exclusively identified in controls (C57BL/6J) and 43 in Townes HbSS. Although no protein remained statistically significant after FDR correction, we selected 268 differentially expressed proteins (DEPs) as candidates (nominal p-value <0.05): 63 upregulated and 205 downregulated in sickle cell mice compared with controls (Supplementary Material 1). The heatmap illustrates the expression profile of DEPs in each sample (Figure 6A). Clustering of experimental groups is shown in the Principal Component Analysis (PCA) graph (Figure 6B). Finally, the distribution of proteins according to the up or downregulation profile is depicted in Volcano plot (Figure 6C).

**FIGURE 6:**
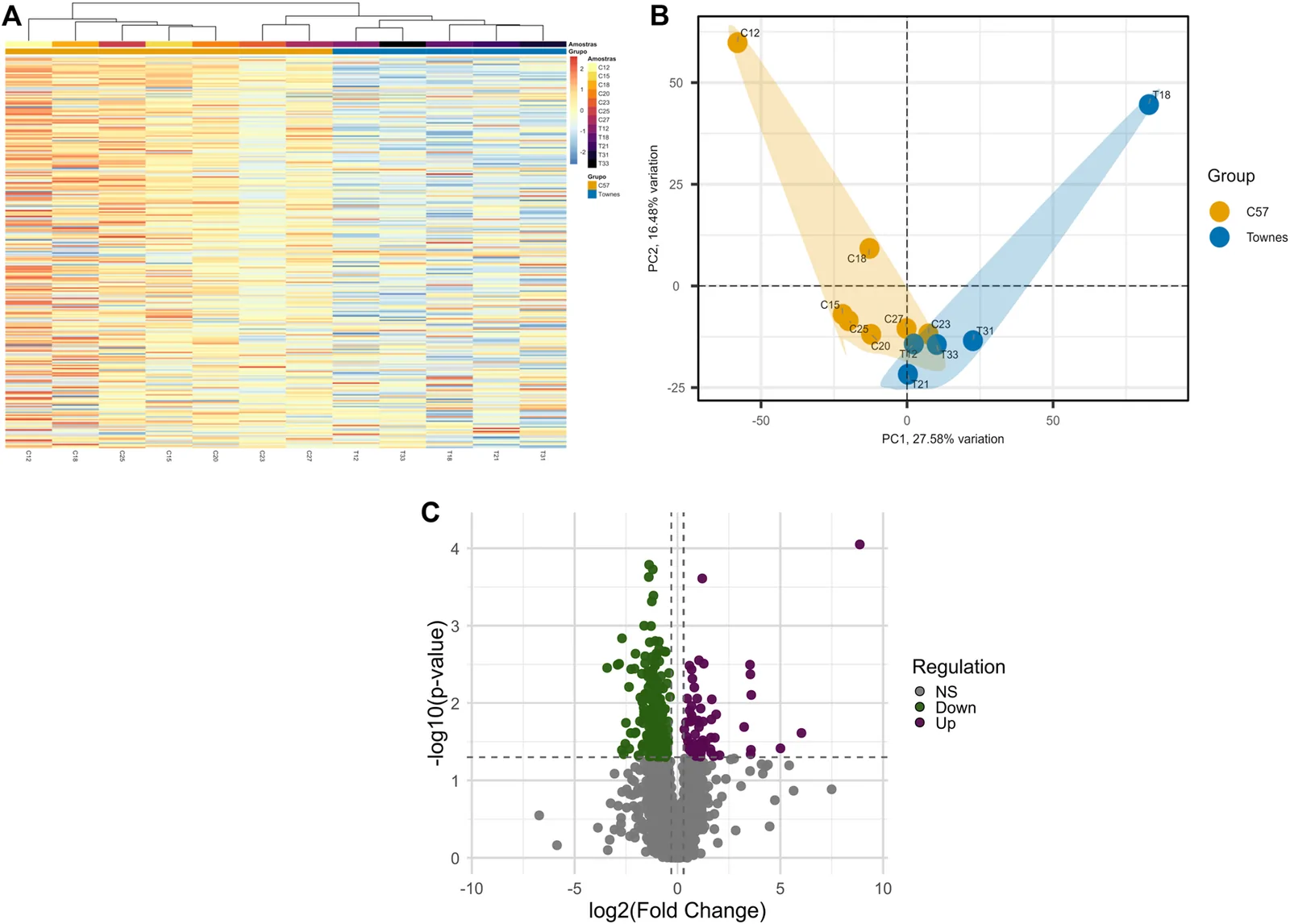
Proteomic analysis graphs. **(A)** Heatmap of DEPs. Rows indicate proteins with significant differences in expression between the two groups; columns represent individual samples. Color intensity is proportional to protein expression. Upregulated proteins are in orange, and downregulated proteins in blue. Clustering method: Euclidean distance. **(B)** Principal Component Analysis (PCA) of samples obtained from the DEPs. Principal Component 1 (PC1) represents 27.58% of group variation, and Principal Component 2 (PC2), 16.48%. Controls (C57BL/6J) are in orange, and Townes HbSS in blue. **(C)** Volcano plot of Townes HbSS versus C57BL/6J comparison. X axis corresponds to Log2 fold change, and Y axis to − log10(p-value). Upregulated DEPs are represented by purple dots, and downregulated DEPs by green dots.

### Pathways of energetic metabolism and phototransduction are enriched in Townes HbSS mice retinas

Functional enrichment analysis was conducted to identify molecular pathways associated with the DEPs in Townes HbSS retinas. Enrichment analysis of downregulated proteins identified 76 molecular pathways, mainly related to phototransduction, energetic metabolism (citric acid cycle and oxidative phosphorylation), and neurodegenerative processes (Figure 7A). For the upregulated proteins, 21 molecular pathways are associated with neurodegenerative processes, glycolysis/gluconeogenesis, and HIF-1 (Hypoxia-Inducible Factor 1) signaling pathway, as shown in Figure 7B.

**FIGURE 7:**
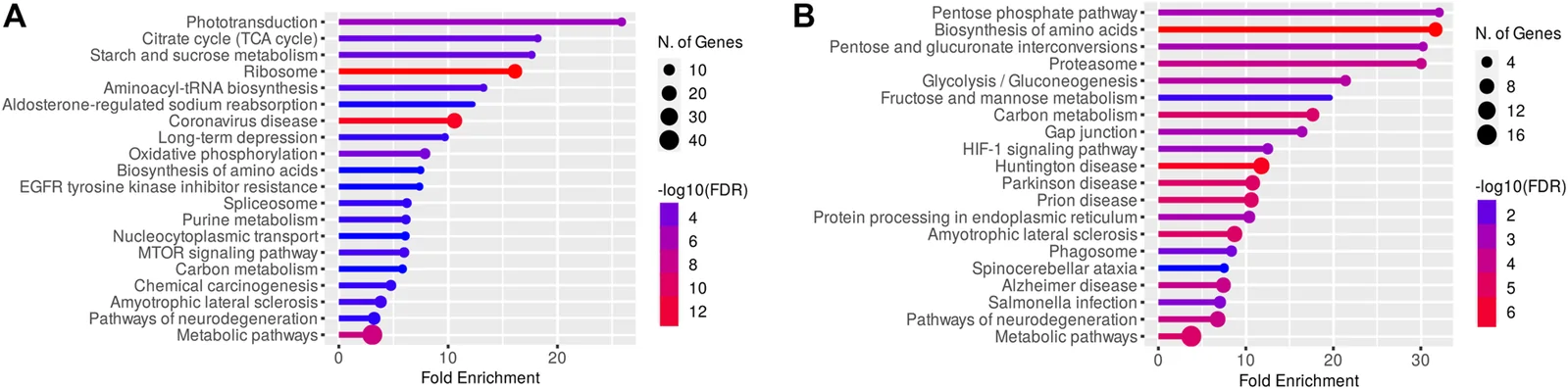
Barplot of the main KEGG molecular pathways enriched from **(A)** downregulated and **(B)** upregulated DEPs. FDR: false discovery rate.

The following analysis was based on pathways related to downregulated DEPs. Protein–protein interaction networks were constructed based on proteins present in each biologically relevant molecular pathway. The pathways selected from the downregulated DEPs were: Phototransduction (Cnga1, Pde6b, Pde6g, Slc24a1, Pde6a, Grk1); Citrate cycle (Aco2, Idh3b, Pcx, Sucla2, Ogdhl); and Oxidative phosphorylation (Ndufb11, Atp5a1, Atp6v1e1, Cox6c, Atp5o, Atp6v1g2, Atp6v1g1, Ndufa7, Uqcrfs1, Uqcrc2) (Figures 8A,B). Additionally, to obtain a more comprehensive view of the molecular profiles of selected targets, differential gene expression analysis was performed. We selected the *Slc24a1* and *Cnga1* genes due to their biological function and relevance in the phototransduction cascade. Both genes are downregulated in Townes HbSS retinas compared to C57BL/6J, in accordance with the protein expression profile observed in the proteomic analysis (Figure 8C).

**FIGURE 8:**
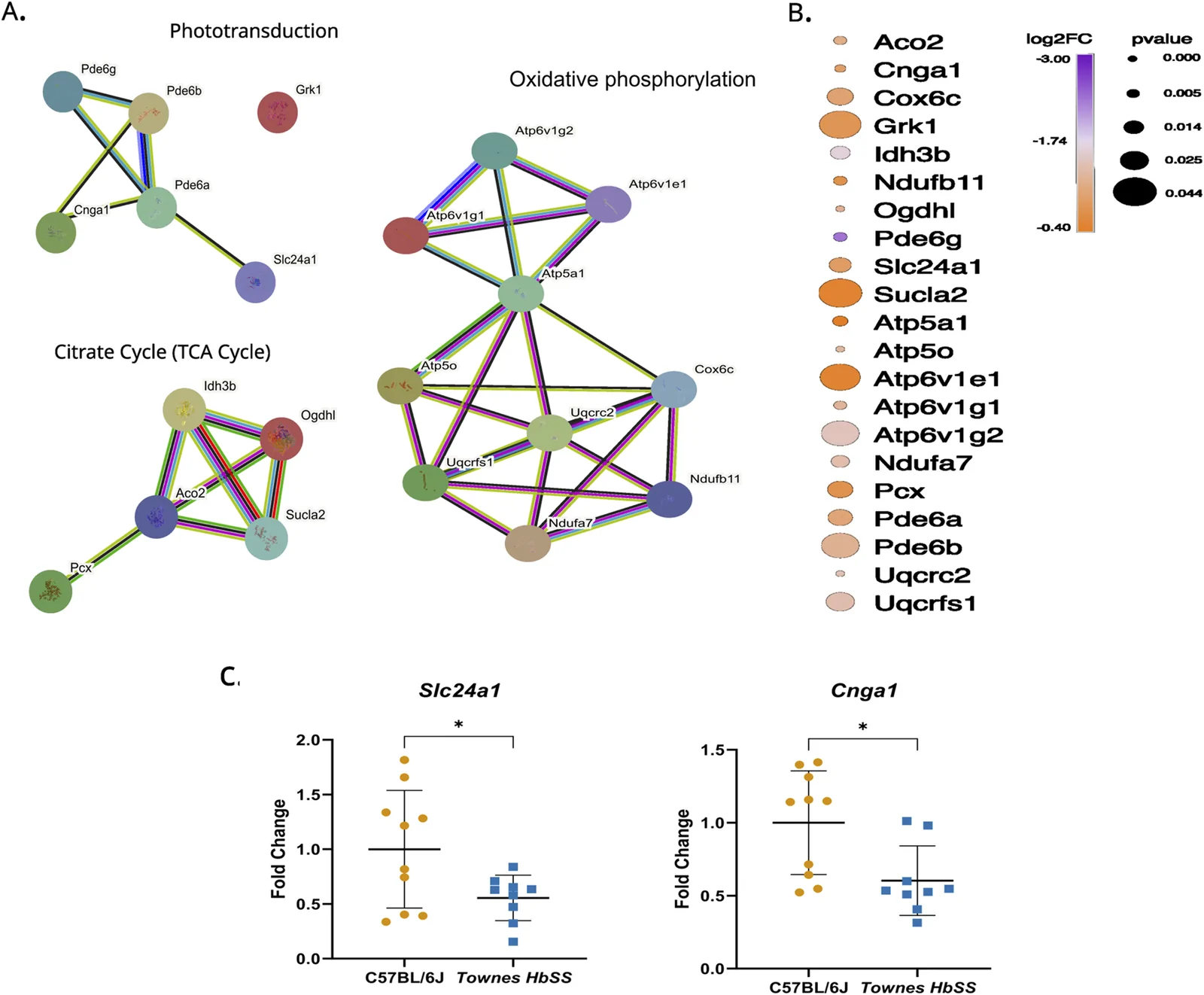
**(A)** PPI network of downregulated proteins presents in the selected pathways. **(B)** Bubble plot of DEPs in the PPI network. The size of the bubble corresponds to the p-value, and the color to the log2FoldChange. **(C)** *Slc24a1* and *Cnga1* gene expression in Townes HbSS compared to C57BL/6J. Student’s t-test. *p < 0.05.

## Discussion

Sickle cell retinopathy is the most important ocular manifestation in SCD. The complex pathophysiology encompasses microvascular occlusion, hypoxia and neovascularization in advanced stages, with retinal detachment and vision loss in 10–20% of the affected eyes [6]. Despite the clinical importance, treatment options are supported by other proliferative retinopathies, with a lack of studies in the SCA context [5]. This is the first study to evaluate the proteomic landscape in the retinas of Townes HbSS, a humanized mouse model of SCA.

The Townes mice are a well-established model for pre-clinical studies in this hemoglobinopathy. Extensive characterization of these mice showed similarities with the human disease phenotype [20, 21]. In accordance with the previous studies, the present experimental group presented expected SCA hematological and metabolic parameters, such as reduction in erythrocyte count, hemoglobin concentration and hematocrit, as well as leukocytosis, reticulocytosis and elevated total, direct and indirect bilirubin, confirming the disease clinical manifestations in our experimental group.

Retinal activity and morphology were assessed to characterize retinopathy. In our ERG results, a significant reduction in a-wave amplitude was observed at the scotopic maximum response, as well as an increase in the implicit time of the photopic b-wave in Townes HbSS. In Promsote et al. [22], these animals, aged between 4 and 7 months, showed reduced amplitudes in the scotopic response for both a- and b-waves, indicating decreased activity, with reduced cellular activation and recruitment in response to the light stimulus. Townes HbSS mice also presented a reduced amplitude at OPs maximum light intensity. This finding may indicate early inner retinal damage, as observed in ischemic retinal conditions such as diabetic retinopathy [23] and retinopathy of prematurity (ROP) [24]. In addition, alterations of the inner retina of Townes HbSS may be reinforced by the qualitative evaluation of the PhNR. Clinically, reductions in this response are related to the retinal ganglion cells activity [25], and the reduction in this post-synaptic neural signal may precede broader ERG changes of the a- and b-waves. To further investigate retinal structural alterations, a histological evaluation was performed. In a qualitative evaluation of H&E sections, C57BL/6J and Townes HbSS showed a less organized and more scattered ONL, indicating photoreceptor involvement in both groups. In Ferdous and colleagues (2021), retinal activity was evaluated in C57BL/6J mice aged between 2 and 32 months. The authors revealed a gradual degeneration of photoreceptors beginning at 6 months of age, with morphological findings of reduction in the overall retinal thickness and in the ONL on Optical Coherence Tomography (OCT) [26]. However, Townes HbSS also presented discrete alterations in the INL, suggesting a distinct pathophysiological mechanism of retinal impairment between groups. Retinal degeneration in C57BL/6J can occur due to aging, and even though age is described as a risk factor for SCR, the retinal impairment observed in Townes HbSS is mainly related to vaso-occlusion and hypoxia.

This hypothesis is mainly supported by the molecular findings of the comparative proteomic evaluation. Upregulation of pathways related to the Hypoxia inducible factor 1 (HIF-1) signaling and neurodegenerative diseases indicates a molecular response due to vaso-occlusion and hypoxia, and a possible impairment of the neural retinal components, as indicated by the inner retina disruptions observed in our HE sections and reduction in the OPs amplitude. Furthermore, pathways of phototransduction and mitochondria-related pathways are downregulated. The retina is one of the most metabolically active tissues, due to the high neuronal energy demand supplied by adaptive vascular networks. The neurovascular units (NVU) comprise vascular, glial and neural elements, and this arrangement guarantees the optimal oxygen and nutrient supplies needed for phototransduction and neuronal activity. In healthy retinas, energy is produced by two main mechanisms: oxidative phosphorylation, an oxygen-consuming pathway associated with higher ATP production; and aerobic glycolysis, in which glucose is converted to lactate as a main ATP source even in the presence of oxygen and elevated mitochondrial respiration [3]. This may occur due a faster ATP production and increased production of carbon for further biosynthetic pathways.

Thus, the vaso-occlusion and hypoxia of retinal microvasculature in SCR directly impact the photoreceptor and neuronal metabolic activity. In a study from Yagi and colleagues, proteomic analysis in a mouse model of retinopathy of prematurity (ROP), another model of proliferative retinopathy, showed that pathways of mitochondrial activity, such as aerobic electron transport chain and oxidative phosphorylation are enriched in the downregulated proteins at the neovascularization peak. Furthermore, the authors confirmed through functional assays that there is a decrease in mitochondrial respiration during neovascularization, and that providing metabolic substrates during reduced mitochondrial function may diminish pathological angiogenesis [27]. Indeed, the same authors confirmed that serine treatment during neo vessel formation in the same ROP mouse model reduced pathological angiogenesis, and led to enrichment of the oxidative phosphorylation process among upregulated proteins [28]. In our results, pathways of TCA cycle and oxidative phosphorylation were enriched in downregulated proteins. Together, these findings suggest that the metabolic alterations in Townes HbSS retinas reflect a molecular adaptation to the hypoxia and vaso-occlusion characteristic of SCR.

Among the downregulated proteins, the phototransduction pathway was also enriched, in which we highlight the Pde6 family (Pde6a, Pde6b and Pde6g), Cnga1 and Slc24a1. The phototransduction process is characterized by light detection by photoreceptors in the retina and the subsequent transduction of the photonic signal into an electrical response. Briefly, photons are absorbed by photopigments present in the outer segment of photoreceptor discs, and the chromophore 11-cis-retinal is isomerized to all-trans-retinal, activating the opsin and subsequently the intracellular messenger transducin. Activated transducin activates in turn phosphodiesterase (Pde6), which hydrolyzes cyclic GMP (cGMP), leading to a decrease in intracellular cGMP levels. As a result, there is a closure in cGMP-gated sodium (Na+) and calcium (Ca^2^+) channels, with consequent hyperpolarization of the photoreceptor membrane and a reduction in glutamate release at the synaptic terminal, thereby transmitting the light signal to downstream retinal neurons [29, 30].

The proteins Pde6a, Pde6b and Pde6g are subunits of the rod phosphodiesterase enzyme. The alpha and beta chains, codified by *PDE6A* and *PD6B* genes, respectively, are the catalytic subunits, regulated by the gamma chain (*PDE6G*). Variants in *PDE6A* and *PD6B* are related to the autosomal recessive form of Retinitis Pigmentosa (RP), the most common cause of inherited blindness characterized by photoreceptor degeneration [31]. Although rare, variants in the *PDE6G* are also described, with patients presenting an early-onset disease [32]. The CNGA1 (Cyclic Nucleotide Gated Channel Subunit Alpha 1) protein is the alpha unit of rod CNG channels, a tetramer composed of three alpha and one beta subunit. This channel is responsible for Na+ and Ca^2^+ influx that depolarizes the photoreceptor and promotes glutamate release in the synaptic terminals. Light stimuli activate the cascade, resulting in the closure of CNG channels and hyperpolarization. Variants in the *CNGA1* gene are also related to autosomal recessive RP [33].

Finally, SLC24A1 (Solute Carrier Family 24 Member 1) is a Na+/Ca^2^+ and K+ exchanger, with Ca^2^+K+ efflux and Na + influx. Thus, this protein is an important regulator of intracellular Ca^2^+ concentrations, regulating rod recovery from light stimuli. Variants in the *SLC24A1* gene are related to autosomal recessive congenital stationary night blindness [34] and RP [35]. In a single-cell RNA-seq analysis, the Pde6g, Cnga1, and Slc24a1 genes were downregulated in a mouse model of RP. According to the authors, this gene expression profile indicates early rod degeneration, prior to clinical manifestations [36].

To validate the molecular pattern of some of the main proteins, we performed RT-qPCR, which showed that the *Cnga1* and *Slc24a1* genes are also downregulated in Townes HbSS mice. It is known that gene expression profiles do not necessarily translate into protein expression, and validation through western blotting or immunofluorescence would be better suited. However, proteomic data combined with RT-qPCR of selected targets indicate downregulation in both gene and protein expression profiles. Thus, the downregulation of Pde6a, Pde6b, Pde6g, Cnga1 and Slc24a1 in Townes HbSS retinas compared to controls may indicate an impairment of phototransduction cascade caused by hypoxia and vaso-occlusion observed in SCR, leading to retinal degeneration.

The lack of validation of the protein expression profile of selected targets and the use of C57BL/6J strain as controls are some of the limitations of the present study. The Townes HbAA strain is the ideal control for the Townes HbSS study model, since it expresses human HbA. However, despite the expression of only murine hemoglobin, the C57BL/6J is the background for Townes model construction, which may reduce the intrinsic strain variability. Another restraint of our study is the lack of retina flat mount, a well stablished protocol for pathological angiogenesis evaluation in proliferative retinopathies. In contrast, retinal alterations due to the sickle cell disease phenotype in Townes HbSS were evident through morphological and, more importantly, the molecular findings, with the identification of relevant pathways and potential targets.

Currently, there are no therapies for NPSCR [37]. PSCR treatment options are laser photocoagulation, anti-VEGF intravitreal injections and vitrectomy in severe cases of vitreous hemorrhage and retinal detachment. However, theses therapies are invasive, with potential side effects, risk of neovascularization recurrence and lacking prospective clinical trials specifically in SCR context [5]. Indeed, anti-VEGF has been shown to be effective in regression of *seafan* vessels and vision improvement [38]. In contrast, Xu and colleagues evaluated the transcriptome and proteome of a mice model for retinal vascular disease with permanent expression of VEGF antibodies. The authors discussed that despite the beneficial effects of VEGF inhibition in vascular regression, this treatment was unable to rescue expression of phototransduction-related genes, which corresponded to reduced photoreceptor cell numbers evaluated by immunofluorescence [39]. Thus, the importance of new therapeutic strategies is emphasized.

To our knowledge, this is the first study to perform a comprehensive evaluation of the retinal proteome in the SCR humanized mouse model. Proteomic analysis has shown reduced abundance of proteins in phototransduction and metabolic pathways, indicating early photoreceptor and energetic alterations prior to more robust and visible morphological and functional changes. The identification of these altered pathways and new targets may, in further studies, expand the therapeutic options, in order to provide a more effective and early treatment strategy to this vision threatening SCD complication.

In conclusion, proteomic analysis in retinas of a humanized mouse model of sickle cell anemia identified differentially expressed proteins in pathways related to sickle cell retinopathy molecular mechanisms, which may enable the identification of therapeutic targets.

## Data Availability

The datasets presented in this study can be found in online repositories. The names of the repository/repositories and accession number(s) can be found in the article/Supplementary Material.
